# An Investigation on Social Representations: Inanimate Agent Can Mislead Dogs (*Canis familiaris*) in a Food Choice Task

**DOI:** 10.1371/journal.pone.0134575

**Published:** 2015-08-04

**Authors:** Judit Abdai, Anna Gergely, Eszter Petró, József Topál, Ádám Miklósi

**Affiliations:** 1 Department of Ethology, Eötvös Loránd University, Budapest, Hungary; 2 Institute of Cognitive Neuroscience and Psychology, Hungarian Academy of Sciences, Budapest, Hungary; 3 MTA-ELTE Comparative Ethology Research Group, Budapest, Hungary; University of Portsmouth, UNITED KINGDOM

## Abstract

The nature of mental representation of others plays a crucial role in social interactions. Dogs present an ideal model species for the investigation of such mental representations because they develop social ties with both conspecifics and heterospecifics. Former studies found that dogs’ preference for larger food quantity could be reversed by humans who indicate the smaller quantity. The question is whether this social bias is restricted to human partners. We suggest that after a short positive social experience, an unfamiliar moving inanimate agent (UMO) can also change dogs’ choice between two food quantities. We tested four groups of dogs with different partners: In the (1) *Helper UMO* and (2) *Helper UMO Control* groups the partner was an interactive remote control car that helped the dog to obtain an otherwise unreachable food. In the (3) *Non-helper UMO* and (4) Human partner groups dogs had restricted interaction with the remote control car and the unfamiliar human partners. In the *Human* partner, *Helper UMO* and *Helper UMO Control* groups the partners were able to revert dogs’ choice for the small amount by indicating the small one, but the *Non-helper UMO* was not. We suggest that dogs are able to generalize their wide range of experiences with humans to another type of agent as well, based on the recognition of similarities in simple behavioural patterns.

## Introduction

The nature of mental representation of others (social representations), for example, whether an animal can accept another agent as a social partner, is a crucial question for those interested in the organisation of social interactions. Thus the relative contribution of genetic and environmental information to the development and management of social representations is an important question for research. Dogs present an ideal model species for studying the relative contribution of genetic and environmental factors because they develop social ties with both conspecifics and heterospecifics (humans) and have been selected for living in an anthropogenic environment. In other words, the basic question is whether genetic changes during domestication enable specific social-cognitive representation of humans in the dogs mind or alternatively, these changes only allow dogs to form more flexible and general social-cognitive representations per se (e.g. [1]). Moreover, others have critised both above suggestions and argued that mental representations of humans are the result of individual experience and genetic influences did not contribute to their nature at all [2].

Here, we propose that dogs have been selected for being able to rely on flexible mental representations of social partners, and their social bias to human-derived social and communicative cues is not specific to a human partner. This flexible social-cognitive representation together with the extensive experience with broad manifestations of human social behaviour on everyday basis leads to a dynamic social representation of companions in the dogs’ mind that allows for rapid generalisation and recognition if a novel, unfamiliar partner shows familiar manifestations of social behaviour. Apart from the fact that such dynamic social-cognitive representation may also be advantageous for dogs who engage with humans in various social interactions on a daily basis, it is also assumed that the recognition of social behaviour is also important to categorise the partner independent from its embodiment.

We suggest that a short experience with an unfamiliar inanimate agent triggers social bias in dogs if that agent displays some behaviour which could be familiar for the dog based on its earlier experience. Gergely et al [3] used an UMO (Unidentified Moving Object—a remote controlled car) having a different embodiment from that of a human or a conspecific to investigate whether dogs show different behaviours toward agents based on their behaviour. They found that if the UMO possessed social-like features, dogs tended to interact with it, and they looked longer at this type of UMO than at a human who showed mechanistic behaviour. This result suggests that in a social interaction the behaviour of the agent is more important than its embodiment, even in a comparison between an unfamiliar inanimate agent and a human partner.

Prato-Previde et al [4] introduced a simple testing procedure in which humans’ ability to influence the dogs’ choice can be measured. They showed that dogs’ preference for large quantities of food could be reversed by humans who indicate the alternative, smaller quantity if they display various forms of expressive behaviours [4–6]. In these studies (e.g. [4]) dogs were typically allowed to make free choices between a larger and a smaller food quantity during 6 trials that were followed by trials in which a human partner indicated the smaller food quantity. In Prato-Previde et al [4] and Marshall-Pescini et al [5] the human partner (either the dogs’ owner or an unfamiliar experimenter) used the same behavioural expressions, i.e. showed his/her interest toward the food quantity by picking up a piece of food while using an enthusiastic tone of voice. It was found that the humans’ familiarity did not influence dogs’ choice. Marshall-Pescini and colleagues [6] were interested about whether the different communicative and expressive behaviours displayed by the partner have an effect on dogs’ choice behaviour. The analysis of the human effect showed that dogs’ choice was influenced by the partner mostly if she picked up the food and brought it to the level of her mouth (with or without using gaze alternation and vocal cue). Authors involved in these studies [4, 5, 6] suggested that dogs are sensitive to human communicative cues and behaviour even if those are misleading. However, as the results of Marshall-Pescini et al [6] show dogs’ counterproductive choice is likely to emerge because of a social bias that can be induced mainly by a salient social cue or a combination of communicative cues.

In connection with these findings an interesting question arises, whether the emerging social bias in family dogs is restricted only to humans (as their natural social partners) or it can be induced by another agent as well.

In the present study we investigated whether an UMO is able to change dogs’ primary choice preference between two different food quantities similarly to humans in former studies [4–6], and whether this is influenced by prior positive experience with the UMO. We copied the procedure reported by Marshall-Pescini et al [6] as far as possible, but in order to be able to compare dogs’ behaviour in the case of a human and a UMO partner, we slightly modified the way how the partners indicated the ‘appropriate’ food quantity. The UMO is not able to display gaze alternation thus the human partner did not show this behaviour either. Because in the case of the UMO we could only use a beeping sound as an attention getting cue, we modified the human partner’s vocal cue from ‘*Oh wow*, *this is good*, *this is so good*!’ to ‘*Hmmm*!’, but kept the friendly tone of voice (for details see Methods). Our hypotheses were that (a) despite of the modified indication the unfamiliar human partner can change dogs’ choice, while (b) after a restricted prior interaction with the UMO dogs would not change their primary choice preference between the food quantities. We predicted that if the UMO helps the dogs to obtain an unreachable food, it can have similar effect on their choice behaviour than a human partner. In order to exclude the possibility that dogs simply associate the food with the beeping sound of the UMO or the UMO itself in the latter group, we created a control group in which we decreased the temporal and spatial contiguity between these.

## Methods

### Ethics statement

Our experiment is based on non-invasive procedures for assessing dogs’ behaviour. Non-invasive studies on dogs are currently allowed to be done without any special permission in Hungary by the University Institutional Animal Care and Use Committee (UIACUC, Eötvös Loránd University, Hungary). The currently operating Hungarian law ‘‘1998. évi XXVIII. Törvény”—the Animal Protection Act–defines experiments on animals in the 9^th^ point of its 3^rd^ paragraph (3. 1/9.). According to the corresponding definition by law, our non-invasive observational study is not considered as an animal experiment.

The owners filled out a consent form to voluntarily permit their dogs to participate in the present study, and that the resulting media can be used in publications. The human partner signed a consent form to participate in the present study and that the resulting media and other data can be used for research purposes.

### Subjects

108 adult pet dogs from different breeds were recruited from the Family Dog Database of the Department of Ethology, Eötvös Loránd University. We excluded 14 dogs because they showed strong side bias (i.e. they approached the same plate in each trial; *Human partner* group: 3 dogs, *Non-helper UMO* group: 5 dogs, *Helper UMO* group: 4 dogs, *Helper UMO Control* group: 2 dogs) and 12 dogs due to procedural problems (e.g. the places of the two food quantities were not counterbalanced or the partner indicated the wrong food quantity; *Non-helper UMO* group: 4 dogs; *Helper UMO* group: 5 dogs; *Helper UMO Control* group: 3 dogs). We excluded two dogs because the owner influenced the dog’s choice (*Helper UMO* group: the owner pushed the dog to one direction several times; *Helper UMO Control* group: the owner pointed to one of the plates several times). We excluded one dog, because the dog did not finish the test (due to the loss of motivation; *Helper UMO Control* group). The remaining 79 dogs constitute four groups as follows: *Human partner* (N = 17; 7 males, 10 females; mean age±SD 4.06±2.38 years), *Non-helper UMO* (N = 22; 9 males, 13 females; mean age±SD 3.41±2.45 years), *Helper UMO* (N = 23; 13 males, 10 females; mean age±SD 4.92±3.4 years) and *Helper UMO Control* (N = 17; 11 males, 6 females; mean age±SD 4.5±2.93 years). Dogs’ age did not differ between groups (Kruskal-Wallis test: $\chi_{3}^{2}$ = 2.1, *p* = 0.55).

Groups were balanced for subjects who chose either the smaller or the larger amount of food more often (15 subjects/groups) in Phase 1 (during the free choice—for details see Procedure). In the *Human partner*, *Helper UMO* and *Helper UMO Control* groups 11 dogs chose the larger, 4 dogs chose the smaller food quantity more often, and 2/8/2 dogs (respectively) chose equally often between them. In the *Non-helper UMO* group 10 dogs chose more often the larger, 5 dogs the smaller food quantity more often and 7 dogs chose equally often between them.

We only tested dogs who could be motivated by food based on the owner’s report. Each subject participated only in one group.

### Apparatus

Dogs were tested at the Department of Ethology, Eötvös Loránd University in a 3 m x 5 m test room. Each trial was recorded by three or four cameras.

**Fig 1:**
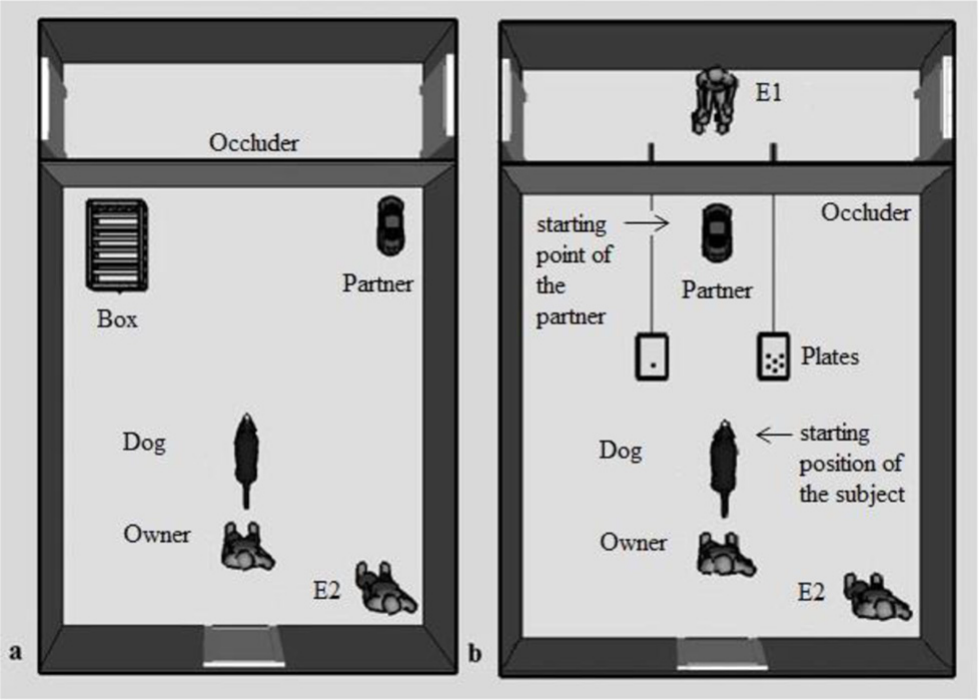
The experimental set up. (a) Phase 2 in the Helper UMO and Helper UMO Control groups, (b) Phase 3: the partner was either a human (E2) or the UMO. The experimental set up in Phase 1 was the same as in Phase 3, but without the presence of the partner. The drawing is not to scale.

To avoid the experimenter’s (E) influence on dogs’ choice, E1 moved the plates (25 cm x 40 cm) by the means of plastic strips from behind an occluder (2 m x 3 m), which remained set up during the whole test. From the dogs’ point of view the plates moved autonomously. The small food quantity meant one piece of sausage and the large food quantity meant six pieces of sausages (see Fig 1B).

### Test partners

In the *Human partner* group the partner was a female human (E2) who used the ‘*Hmmm*!’ sound in a high pitched friendly voice as an attention getting cue. In the *Non-helper*, *Helper* and *Helper UMO Control* groups we used a remote control car (#7304 Traxxas 1/16 Ford Mustang Boss 302; 37 cm x 18.5 cm x 12 cm) as a partner with a magnet on its front. UMO emitted a high pitched beeping sound (3200 Hz) as an attention getting cue. UMO’s movements and sounds were remotely controlled by E2.

### Procedure

#### Phase 1

After the dog explored the room, the owner sat on a chair and held the dog in front of him/her. The trial started when the dog was at his/her starting position. Upon E2’s verbal indication (‘*Okay*!’) E1 (who hid behind the occluder) pushed simultaneously both plates with the different food quantities to their predetermined location (Fig 1B). After the dog oriented its head toward both the plates at least once the owner released the dog on the signal of E2. E1 pulled back the non-chosen plate before the dog could reach it. A helper from outside the room or E2 indicated E1 which plate should be removed, by saying the side of the plate from E1’s viewpoint. Then the owner called the dog back and the whole procedure of the choice trial was repeated (altogether six trials). The sides of the small and large food quantitites were counterbalanced (RLLRRL/LRRLLR). After the last trial the owner and the dog left the room for 2 minutes. This phase was the same for all dogs.

#### Phase 2

*Human partner group*: The owner and the dog entered the room with E2. The dog was free to move in the room for 2 min, while E2 was talking with the owner and petted the dog. The human partner petted the dog depending on its willingness (i.e. if, and only if that the dog approached her and initiated physical contact). This was so because both unresponsive (who refuse to pet the dog) as well as intrusive (petting the dog who do not like being petted) human partners could trigger negative emotions. However, all subjects were petted at least once by the human partner when they approached her within 0.5 m. If they did not approach her, after 30 s she stepped to the dog when (s)he was within 2 m and petted him/her.

*Non-helper UMO group*: Before the owner and the dog came back, E2 placed the UMO to the middle in front of the occluder. The owner sat on the chair and held the dog in front of him/her. E2 stood in the corner on the right side of the dog. The UMO moved around the room for 2 min, after the time elapsed, it stopped at the starting point. In the first round the UMO stopped in front of the dog for a few seconds, so the dog could look at it and smell it from up close.

*Helper UMO group (similar to [3])*: Before the owner and the dog came back, E1 placed a wire mesh box (61 cm x 46 cm x 54 cm) to one side of the room next to the occluder and E2 placed the UMO to the other side of the room next to the occluder (Fig 1A). The owner sat on the chair and held the dog in front of him/her. E2 stood in the corner on the right side of the dog. E1 called the dog’s attention (‘*Hi (Dog’s name)*, *look*!’) with a piece of food in her hand, put it on a plastic plate which had metal sheets on its sides and attached the plate to a magnet inside the box. Then she left the room and the dog was free to move in the room and could try to get the food for 15 s. After the time elapsed E2 called the dog’s attention by making the UMO beep and move into the box, where it emitted the beeping sound again when it attached to the plate. The UMO brought out the plate, transported it to the dog who ate the food. E1 entered, put the box to the other side of the room and the UMO went to the former place of the box (i.e. the place of the box and the starting point of the UMO were switched in every trial). The above described procedure was repeated a total of six times. From the 2^nd^ trial at any time when the freely moving dog looked at the UMO, the partner started to move immediately and brought out the plate. In this case we used the beeping sound only when the UMO attached to the plate inside the box.

*Helper UMO Control group*: The procedure was similar to that applied in the *Helper UMO* group. However, we called the dogs’ attention with the beeping sound only after the UMO started to move, during the first half of the route to the box in every trial. Thus the beeping sound in this phase was never emitted next to the food or even in the proximity of the cage. The UMO took the food to the dog and stopped about 50 cm away from the dog. Then the owner removed the plate from UMO and handed the food over to the dog.

After the last trial the owner and the dog left the room for 2 minutes.

#### Phase 3

In each group the respective partner indicated the food quantity opposite to the primary preference of the dog (which the dog initially chose at least four times out of six) in *Phase 1*. If the dog chose equally often between the two food quantities, the partner indicated the smaller one.

*Human partner group*: The owner and the dog came back to the room, and the owner sat on the chair and held the dog in front of him/her. The human partner (E2) stood at the starting point, in the middle next to the occluder. E1 pushed the plates with the different food quantities to their predetermined location from behind the occluder (Fig 1B). The partner went to the appropriate quantity, squatted down, lifted up one piece of food to the level of her mouth and said ‘*Hmmm*!’ in a high pitched friendly voice. Then she put the food back and stood at the starting point with her back turned to the subject. The owner released the dog that could choose between the plates. E1 pulled back the non-chosen plate before the dog could reach it. The owner called the dog back and the partner turned toward the dog again. During Phase 3 the partner did not make any eye contact with the dog.

*Non-helper UMO*, *Helper UMO* and *Helper UMO Control groups*: Before the owner and the dog came back E2 placed the UMO to the starting point. The owner sat on the chair and held the dog in front of him/her. E2 stood in the corner on the right side of the dog. The procedure differed from the *Human partner* group only in how the partner indicated one food location. UMO moved to the appropriate quantity, and then it stopped directly behind the food, it sounded the beep to call the dog’s attention and moved back to the starting point.

We repeated the above described procedures a total of six times in each group. The sides of the small and large food quantitites were counterbalanced.

Dogs that started with the first sequence (RLLRRL) in Phase 1, were tested with the second sequence in Phase 3 (LRRLLR) and vice versa.

### Behavioural variables and data analysis

The tests were videotaped and dogs’ behaviour was analyzed with Solomon Coder 14.03.10. (András Péter http://solomoncoder.com). One videotape was damaged, the choices of this subject were recorded by live coding (*Helper UMO* group, the dog chose equally often between the food quantities in *Phase 1*). Behavioural variables:

Phase 1 and 3:

- *Choice (0/1)*: we scored each trial as 1 if the dog chose to food plate indicated by the partner and as 0 if the dog chose the non-indicated food quantity (we scored each trial in *Phase 1* retrospectively based on dogs’ choice in *Phase 3*; e.g. when the dog chose the large quantity more often in *Phase 1*, the partner indicated the small quantity in *Phase 3*, thus the small quantity was scored as 1 and the large quantity was scored as 0)

Phase 3:

- *Looking at the partner (%)*: looking duration at the partner (s) during the trial (from the appearance of the plates until the choice) / total time of the trial (s) * 100

For statistical analysis we used IBM SPSS Statistics 21.

We used GLMM for Binomial Distribution to test whether the group (*Human partner*, *Non-helper UMO* and *Helper UMO*), the phase (*Phase 1* and *3*) or the *Group* x *Phase* interaction had an effect on dogs’ choice.

We calculated the percent of the choices in *Phase 1* and *3* in all groups and used One-Sample Wilcoxon signed-rank test to compare dogs’ choice to chance level (50%).

We compared the *Looking at the partner (%)* variable between groups with Independent-samples Kruskal-Wallis Test with Dunn post-hoc test. From this analysis we had to exclude ten dogs, because we could not code the looking data of the subjects due to the quality of the recorded videos (*Human partner*: 2 dogs, *Non-helper UMO*: 3 dogs, *Helper UMO*: 2 dogs, *Helper UMO Control*: 3 dogs; the more frequent choice of the large/small quantity in Phase 1: 5/1 dogs respectively, equal choice between the food quantities: 4 dogs).

For the *Looking at the partner (%)* variable 20% of the subjects were coded by a second observer. The Cronbach’s alpha was 0.984.

## Results

In our experiment only 54.4% of dogs chose the large food quantity at least four times out of six, in contrast in previous studies more than 70% of dogs displayed such preference (see S1 Table) [4–6]. Because in the course of this present experiment we cannot determine the casual factors behind the dogs’ choice, the subsequent analysis has been restricted only to dogs that chose the large food quantity more often in Phase 1.

**Fig 2:**
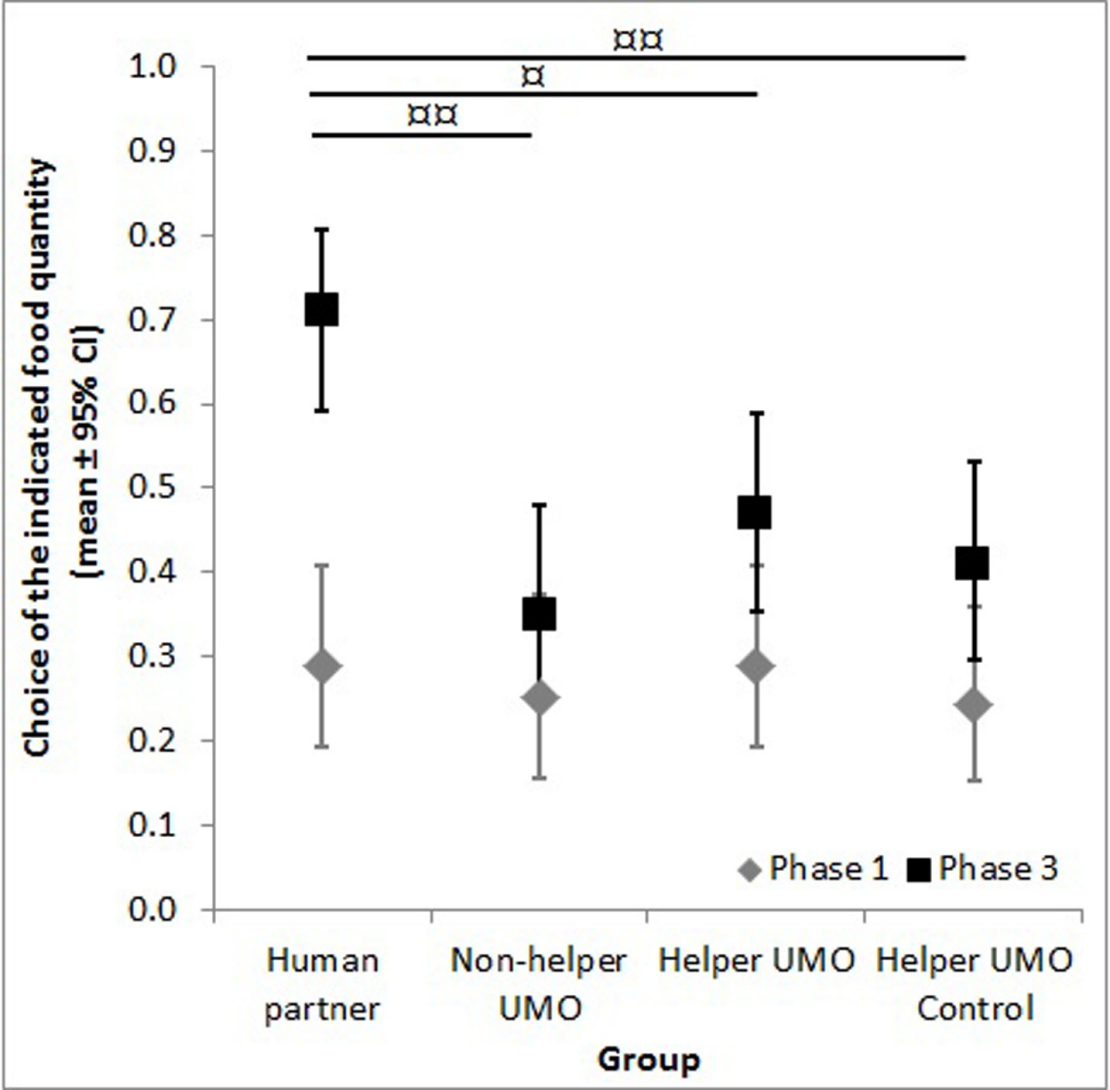
Choice of the small food quantity in Phase 1 and 3. Data are only from dogs who chose the larger quantity more often (Phase 1) and the partner indicated the small food quantity in Phase 3; * shows the difference between phases, ¤ shows the difference between groups in Phase 3 (* p<0.05, ** p<0.001, ¤ p<0.01, ¤¤ p<0.001).

Based on the results both the group and the phase had an effect on dogs’ choice (Group: F_3,508_ = 3.87, *p* = 0.009; Phase: F_1,508_ = 24.92, *p*<0.001), while the group *x* phase interaction was not significant (F_3,508_ = 2.25, *p* = 0.082). The comparison between phases showed significant difference in the *Human partner*, the *Helper UMO* and the *Helper UMO Control* groups (*Phase 1* vs *3*: *Human partner p*<0.001; *Non-helper UMO p* = 0.231; *Helper UMO p* = 0.030; *Helper UMO Control p* = 0.039). In *Phase 1* there was no difference between groups (*Human partner* vs *Non-helper UMO*: *p* = 0.631; *Human partner* vs *Helper UMO*: *p* = 1.00; *Human partner* vs *Helper UMO Control*: *p =* 0.553; *Non-helper UMO* vs *Helper UMO*: *p* = 0.636; *Non-helper UMO* vs *Helper UMO Control*: *p* = 0.921; *Helper UMO* vs *Helper UMO Control*: *p* = 0.553), but in *Phase 3* there were significant differences between the *Human partner* and *Non-helper UMO* groups, the *Human partner* and *Helper UMO* groups and the *Human partner* and *Helper UMO Control* groups (*Human partner* vs *Non-helper UMO*: *p*<0.001; *Human partner* vs *Helper UMO*: *p* = 0.004; *Human partner* vs *Helper UMO Control*: *p<*0.001; *Non-helper UMO* vs *Helper UMO*: *p* = 0.171; *Non-helper UMO* vs *Helper UMO Control*: *p* = 0.497; *Helper UMO* vs *Helper UMO Control*: *p* = 0.483; see Fig 2).

Results of the Wilcoxon test showed that dogs’ choice in Phase 1 was below the chance level in all of the groups, i.e. they chose the large food quantity more often. In Phase 3 dogs’ choice was below the chance level only in the *Non-helper UMO* group, while in the *Human partner*, *Helper UMO* and *Helper UMO Control* groups it was at chance level (*Phase 1*: *Human partner* N = 11, z = -3.071, *p* = 0.002; *Non-helper UMO* N = 10, z = -2.913, *p* = 0.004; *Helper UMO* N = 11, z = -3.071, *p* = 0.002; *Helper UMO Control* N = 11, z = -3.002, *p* = 0.003; *Phase 3*: *Human partner* N = 11, z = 1.801, *p* = 0.072; *Non-helper UMO* N = 10, z = -2.264, *p* = 0.024; *Helper UMO* N = 11, z = -0.707, *p* = 0.48; *Helper UMO Control* N = 11, z = -1.897, *p* = 0.058).

There was a difference between groups in duration of looking at the partner in *Phase 3* (Kruskal-Wallis test: $\chi_{3}^{2}$ = 8.94, *p* = 0.03). The results of the Dunn post-hoc test showed that dogs looked longer at the human than at the helper UMO partner, but there were no significant differences between any other partners (*Human partner* vs *Non-helper UMO*: *p* = 0.392; *Human partner* vs *Helper UMO*: *p* = 0.019; *Human partner* vs *Helper UMO Control*: *p* = 1.000; *Non-helper UMO* vs *Helper UMO*: *p* = 1.000; *Non-helper* vs *Helper UMO Control*: *p* = 1.000; *Helper UMO* vs *Helper UMO Control*: *p* = 0.878).

## Discussion

Our results show that a human partner was able to change dogs’ primary preference to the large food quantity similarly to former studies [4–6]. More importantly we also found that a short positive prior interaction with the UMO changed the dogs’ choice in the same direction. Notably, however, dogs’ choice performance in the *Helper UMO* and *Helper UMO Control* groups does not reach the same level as in the *Human partner* group (see Fig 2). Statistically similar results were found in the overall analysis when all subjects are included independently from their primary choice (see S1 Supporting Information).

In line with our hypothesis dogs have shown social bias only if they had specific social experience with the agent (human or helper UMOs). We suggest that the experience of receiving food directly or indirectly from the UMO triggered the social-cognitive mental representation of humans which made the dog respond to the UMO as it was a social partner. Obviously, the role of associative learning cannot be ruled out because dogs were exposed to a food-UMO constellation six times during Phase 2, and this may bias them to approach the agent for food. In the *Helper UMO Control* group dogs could observe the UMO delivering the food, but the food was actually given to the dogs by the owners. This interference by the owner also decreased the likelihood that the dog would associate the UMO with the food. Furthermore, if the dog had associated UMO with food then we would expect him to approach the agent during the choice test rather the location in space (small amount of food in Phase 3) which was earlier visited by the UMO. Importantly, dogs’ social bias was present from the beginning of Phase 3 and overall only two dogs approached the UMO before choosing between the quantities in the first trial of Phase 3 (for more details see S2 Supporting Information).

It is important to note that in analogous situations dogs fail to learn if the position of the food is indicated by a beacon. For example, Agnetta et al [7] used a marker placed close to hidden food in a two way choice test. In one of the conditions, which was repeated twice (as the first and last condition), the dogs were only able to see the placed marker without a human’s movement. Although dogs were tested in overall 36 trials, they showed no sign of learning.

One might suggests that the mobility and attention getting cue of the UMO was more salient compared to the immobility of the marker. However, note that the marker was clearly visible next to the hiding place throughout the subjects’ choices [7]. Using small sticks as beacons Milgram et al [8] reported that dogs needed a few 100 trials to learn the location of the hidden food. In addition, note that the UMO moved back to the middle position between the two food-patches before the dog made its choice, thus it had to approach the ‘indicated’ place using short term memory.

Thus it seems to be a more parsimonius explanation that the social-cognitive representation, which has been established through diverse interactions with humans, triggered the social bias observed as a response to the Helper UMOs’ action. The feeding and helping interaction with UMOs likely primed social-cognitive mental representations, as these were similar to significant aspects of human-dog interaction. In other words the social bias in the case of the helper UMOs emerged because dogs recognised the similarity between their feeding and helping interaction with the inanimate agent and similar (earlier) interactions with humans. This recognition (correspondence) allowed a rapid change in behaviour without the need for laborious trial and error learning. Importantly, this generalisation occurred toward an agent with different movement/behaviour abilities and embodiment.

Dogs’ domestication history (e.g. [9]) and the wide range of experiences with a heterospecific agent might help to develop a more flexible social behaviour. Although the selective effect of the human social environment cannot be excluded we suggest that social experiences with humans during ontogeny can be sufficient to cause such rapid changes in dogs’ social behaviour. As noted above the flexible utilisation of such social-cognitive mental representation could provide the basis for dogs’ ability to manage certain (quite specific) human features and behaviours (e.g. communicative behaviour, [10]). At the same time this kind of representation may allow for generalisation if an unfamiliar agent (independently from its embodiment) displays human-like behaviours.

Contrary to former studies [4–6] only 54.4% of dogs chose the large food quantity more often during the free choice in this experiment. Thus the possibility emerges that the underlying mechanism in dogs’ free choice is not the ‘*‘more’ is better than ‘less”* ([11], p. 1793) rule. We suggest three alternative explanations: (1) dogs’ choice in the absence of a partner (in subjects that chose any one of the food quantities more often) was due to a true preference toward the particular food quantity and thus following the indication of the partner was a social bias; (2) considering that 24.1% of dogs chose equally between the quantities during the free choice, it might be possible that dogs tried to look for a rule (e.g. which one should they choose), and the indication of the partner helped to understand this ‘rule’; and (3) dogs may have relied on the ‘follow a human’s signal’ rule and generalized it to the partners in this experiment. Topál et al [12] found that dogs tend to follow some kind of social rule in a hide-and-search task, for example ‘following the partner’s route’. Thus at present our second or third suggestions appear to be the most likely explanations.

It may not be totally ruled out that unintended human influence (see ‘Clever Hans effect’; [13]) might have played a role in dogs’ behaviour. This is however unlikely because, first, the plates moved autonomously from the dogs’ viewpoint, thus the only available cue could be the owners’ behaviour, however, they were blind to the purpose of the study. If they had specific expectation, we would have not expected difference in dogs’ bias among the four groups. Second, Hegedűs et al [14] found that in a two-way choice test even the owners pushing their dogs to one side does not influence dogs’ choice. Schmidjell et al [15] also reported that in a similar task the owner’s belief about the purpose of the study does not influence dogs’ choice.

Our results showed that similar change happens in dogs’ choice in the case of the helper UMOs as in the *Human partner* group, but the magnitude of change is not equal. We propose that more experience with the UMO might have led to a more similar result. However, we cannot exclude the possibility, that although dogs’ social behaviour have become more flexible, it only enables them to generalize their social experience with humans to an UMO to a certain level, and the effect of experience with an UMO has its upper limit.

In sum, we suggest that dogs due to their substantial social experience with humans, are able to detect similar behavioural patterns in an other type of agent as well, independently from its embodiment, and thus are able to relate to the agent as a social partner even after just a short positive experience.

## Supporting Information

S1 Table Dogs’ choice in the free choice conditions (Prato-Previde et al, 2008; Marshall-Pescini et al 2011; 2012) and in Phase 1 (present study). (DOCX)

S1 Supporting Information Data analysis of all subjects independently from their prior choice. (DOCX)

S2 Supporting Information The effect of the possible food-UMO association on dogs’ choice. (DOCX)

S3 Supporting Information Detailed information about the subjects and data. (XLSX)
