# Correction: An Investigation on Social Representations: Inanimate Agent Can Mislead Dogs (*Canis familiaris*) in a Food Choice Task

**DOI:** 10.1371/journal.pone.0139531

**Published:** 2015-09-25

**Authors:** Judit Abdai, Anna Gergely, Eszter Petró, József Topál, Ádám Miklósi

There are errors in Fig 2, “Choice of the small food quantity in Phase 1 and 3.” Please view the corrected Fig 2 here.

**Fig 2:**
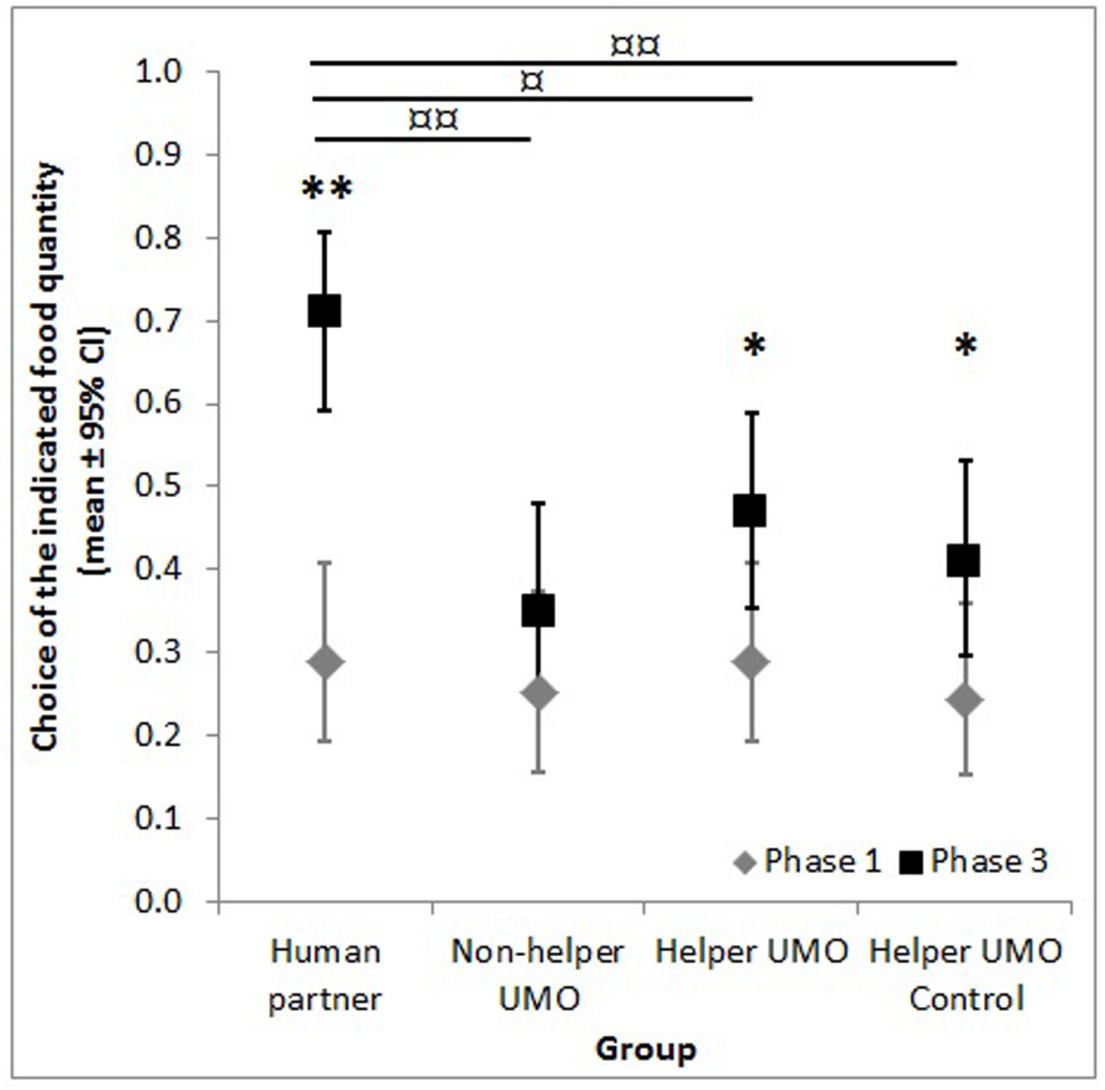
Choice of the small food quantity in Phase 1 and 3. Data are only from dogs who chose the larger quantity more often (Phase 1) and the partner indicated the small food quantity in Phase 3; * shows the difference between phases, ¤ shows the difference between groups in Phase 3 (* p<0.05, ** p<0.001, ¤ p<0.01, ¤¤ p<0.001).
