# Supplementary material for: An Investigation on Social Representations: Inanimate Agent Can Mislead Dogs (Canis familiaris) in a Food Choice Task
Source: PLoS One. 2015 Aug 4;10(8):e0134575. doi: 10.1371/journal.pone.0134575 (PMC4524664; doi:10.1371/journal.pone.0134575)
Supplement: S1 Table — (DOCX) [file pone.0134575.s001.docx]

***Table S1*** *Dogs’ choice in the free choice conditions (Prato-Previde et al, 2008; Marshall-Pescini et al 2011; 2012) and in Phase 1 (present study)*

|  | Large quantity (%) | Small quantity (%) | Equally often (%) |
| --- | --- | --- | --- |
| Prato-Previde et al, 2008 | 74 | 8 | 18 |
| Marshall-Pescini et al, 2011 | 79 | No Information | No Information |
| Marshall-Pescini et al, 2012 | 73 | 4 | 23 |
| Present study | 54.4 | 21.5 | 24.1 |
