# Supplementary material for: An Investigation on Social Representations: Inanimate Agent Can Mislead Dogs (Canis familiaris) in a Food Choice Task
Source: PLoS One. 2015 Aug 4;10(8):e0134575. doi: 10.1371/journal.pone.0134575 (PMC4524664; doi:10.1371/journal.pone.0134575)
Supplement: S1 Supporting Information — (DOCX) [file pone.0134575.s002.docx]

**Data analysis of all subjects independently from their prior choice**

Based on the results the group *x* phase interaction had an effect on dogs’ choice (F_3,940_=5.97, *p*<0.001). The comparison between phases showed significant difference in the *Human partner,* *Helper UMO* and *Helper UMO Control* groups (*Phase 1* vs *3*: *Human partner* *p*<0.001; *Non-helper UMO* *p*=0.206; *Helper UMO p*=0.02; *Helper UMO Control p*=0.026). In *Phase 1* there was no difference between groups (*Human partner* vs *Non-helper UMO*: *p*=0.55; *Human partner* vs *Helper UMO*: *p*=0.288; *Human partner* vs *Helper UMO Control*: *p*=0.535; *Non-helper UMO* vs *Helper UMO*: *p*=0.625; *Non-helper UMO* vs *Helper UMO Control*: *p*=0.207; *Helper UMO* vs *Helper UMO Control*: *p*=0.082), but in *Phase 3* there were significant difference between the *Human partner* and *Non-helper UMO* groups, the *Human partner* and *Helper UMO* groups, and the *Human partner* and *Helper UMO Control* groups (*Human partner* vs *Non-helper UMO*: *p*<0.001; *Human partner* vs *Helper UMO*: *p*<0.001; *Human partner* vs *Helper UMO Control*: *p*<0.001; *Non-helper UMO* vs *Helper UMO*: *p*=0.135; *Non-helper UMO* vs *Helper UMO Control*: *p*=0.94; *Helper UMO* vs *Helper UMO Control*: *p*=0.141). See the results on *Figure S1*.

***Figure S1*** *Choice of the indicated food quantity in Phase 1 and 3. In Phase 3 the partner indicated the opposite food quantity of that chosen by the dog more often in Phase 1; ­* shows the difference between phases, ¤ shows the difference between groups in Phase 3 (* p<0.05, ** p<0.001, ¤ p<0.001)*

Results of the Wilcoxon test showed that dogs’ choice in Phase 1 was below chance level in all groups, i.e. they chose the non-indicated food quantity more often. In Phase 3 dogs’ choice was below the chance level only in the Non-helper UMO group, while in the Helper UMO group it was on chance level and in the Human partner group dogs reversed their choice (*Phase 1: Human partner* N=17, z=-3.542, *p*<0.001; *Non-helper UMO*  N=22, z=-3.571, *p*<0.001; *Helper UMO* N=23, z=-3.626, *p*<0.001; *Helper UMO Control* N=17, z=-3.487, *p*<0.001; *Phase 3: Human partner* N=17, z=2.656, *p*=0.008; *Non-helper UMO* N=22, z=-2.351, *p*=0.019; *Helper UMO* N=23, z=0.097, *p*=0.923; *Helper UMO Control* N=17, z=-1.897, *p*=0.058).

Looking duration at the partner in *Phase 3* varied among groups (Kruskal-Wallis test: N=68, $\chi_{3}^{2}$ =12.64, *p*=0.005). The results of the Dunn post-hoc test showed that dogs looked longer at the human than at the non-helper partner, but there was no significant difference between any other partners (*Human partner* vs *Non-helper UMO*: *p*=0.003; *Human partner* vs *Helper UMO*: *p*=0.066; *Human partner* vs *Helper UMO Control*: *p*=0.349; *Non-helper UMO* vs *Helper UMO*: *p*=0.914; *Non-helper UMO* vs *Helper UMO Control*: *p*=0.936; *Helper UMO* vs *Helper UMO Control*: *p*= 1.000).
