# Supplementary material for: An Investigation on Social Representations: Inanimate Agent Can Mislead Dogs (Canis familiaris) in a Food Choice Task
Source: PLoS One. 2015 Aug 4;10(8):e0134575. doi: 10.1371/journal.pone.0134575 (PMC4524664; doi:10.1371/journal.pone.0134575)
Supplement: S2 Supporting Information — (DOCX) [file pone.0134575.s003.docx]

**The effect of the possible food-UMO association on dogs’ choice**

If the food-UMO association during Phase 2 had an effect on dogs’ behaviour, we expect that they would approach the UMO first in the first trial of Phase 3. However, based on the recorded videos only two dogs approached the UMO before the choice in the first trial of Phase 3 (one of them chose the large, the other one chose the small food quantity more often in Phase 1).

We used GLMM for Binomial Distribution to test whether there is a difference between dogs’ choice in the first and second half of Phase 3. We did not find significant difference between the two halves of Phase 3 in the different groups (all subjects independently from their prior choice: F_3,466_=0.567, p=0.637; dogs chose more often the large food quantity in Phase 1: F_3,250_=0.446, p=0.720). Our result showed no significant difference between the first and second half of the trials in Phase 3 (all subjects independently from their prior choice: F_1,466_=0.000, p=0.984; dogs chose more often the large food quantity in Phase 1: F_1,251_=0.009, p=0.925).
